# On the stability & phase locking to a system reference of an optoelectronic oscillator with large delay

**DOI:** 10.1038/s41598-023-31248-w

**Published:** 2023-03-14

**Authors:** Mehedi Hasan, Charles Nicholls, Trevor Hall

**Affiliations:** 1 Photonic Technology Laboratory, Advanced Research Complex, University of Ottawa, 25 Templeton Street, Ottawa, ON K1N 6X1 Canada; 2 Nanowave Technologies Inc., 6 Gurdwara Rd, Nepean, ON K2E 8A3 Canada

**Keywords:** Optics and photonics, Physics

## Abstract

Delay line oscillators based on photonic components, offer the potential for realization of phase noise levels up to 3 orders of magnitude lower than achievable by conventional microwave sources. Fibreoptic-based delay lines can realize the large delay required for low phase noise systems whilst simultaneously achieving insertion loss levels that can be compensated with available microwave and photonic amplification technologies. Multimode operation is an artefact of the delay line oscillator and introduces modulational instability into phase-locked control loops. An optoelectronic oscillator (OEO) with large delay under proportional integral control by a phase-locked loop (PLL) is modelled, providing the first report of the location of all the infinity of poles of the PLL-OEO system function. The first experimental observation of giant phase modulated oscillation of a free OEO and spontaneous giant phase modulated oscillation of a PLL-OEO are also reported and explained respectively as a source and manifestation of modulational instability. Nevertheless, the analysis and experimental observations, including a prototype 10 GHz PLL-OEO phase noise spectral density achieving $- 80dBc/Hz {\text{at}} 10 Hz$ and $- 145dBc/Hz {\text{at}} 10 kHz$, demonstrate that stable phase lock operation and optimum phase noise performance is achievable provided full account of the multimode nature of the OEO is taken in the phase lock analysis.

## Introduction

The random fluctuations of an oscillator limit the precision of time and frequency measurements on which scientific and technological endeavours rely. The noise and long-term stability of the system oscillator / clock is of major importance in applications such as optical and wireless communications, high-speed digital electronics, radar, and astronomy. With ever-increasing clock frequencies being used in digital systems, the requirement for compact high-performance clock sources will continue. The development of such a source would have major impact, for example, on radar sensitivity through improved clutter rejection; on the generation of mm waves for 5G wireless; and on research into sources of THz radiation.

Among a variety of means using photonics to generate microwaves, the optoelectronic oscillator (OEO) is the most suited to practical deployment. Reference^1^ provides a review of the large literature that has arisen following the introduction in 1996 of the OEO^2^. Lasers and OEOs are examples of time delay oscillators. The laser generates optical carriers using a cavity containing the sustaining amplifier. The OEO generates microwave carriers using an RF photonic link consisting of laser; optical intensity modulator; optical fibre; photo-receiver; RF amplifier and bandpass filter, which drives the modulator; closing the loop and sustaining oscillation. The virtue of time delay oscillators is the large delay achievable relative to the oscillation period. The low loss of optical fibre (0.2 dB/km) permits delay line lengths of ~ 10 km offering exceptional OEO phase noise performance. However, the frequency interval between adjacent oscillation modes becomes very small (20 kHz for 10 km), and filtering is needed for mode selection and sidemode suppression.

Whereas quartz crystal system reference oscillators, even when multiplied to microwave frequencies, offer superior phase noise at close-to-carrier offset frequencies, the OEO offers superior phase noise performance at offset frequencies further out from the carrier. It remains a requirement to phase lock the OEO to the system reference to reduce the close-in phase noise and to provide long-term stability while engineering the phase-locked loop (PLL) to take advantage of the superior phase noise of a free OEO at higher offset frequencies.

A variety of architectures and approaches to locking an OEO to a system reference have been disclosed in the literature^3–8^. In most cases, the PLL is combined with injection locking; either external-injection locking of the OEO to the reference carrier^6^ or self-injection locking of the OEO to a delayed replica of the oscillation^7,8^. The latter category encompasses dual loop OEOs^5^ and more generally multi-loop OEOs as a large self-injection level special case. The theoretical models disclosed to describe these architectural variations neglect the multimode character of the OEO. In respect of injection locking the models reduce to the differential equations of Adler (weak injection)^9^ or Paciorek (strong injection)^10^ valid only for classical single mode oscillator. Recently, a delay integral/differential equation formulation of injection locking theory for a time delay oscillator has been introduced^11^ that fully accounts for the multimode character of the oscillator and the distinct physical roles of the delay line and RF bandpass filter. It is long established that injection locking has an equivalent representation as a type-I PLL^12^, i.e., a proportional controller, so these architectures may be viewed from the perspective of self-referenced phase locked loops, which has been applied to the study of self-injection locked electronic oscillators and a short loop OEO^3^. In respect of the PLL, prior models treat the controlled OEO as a voltage-controlled oscillator (VCO) and consequently reduce to the classical theory in which the VCO is treated as a perfect phase integrator. The multimode operation and long delay are fundamental to the neuromorphic application of a broadband OEO as a reservoir computer^13,14^ but this paper is concerned with the OEO as a source of pristine RF carriers.

A single-loop OEO under proportional integral control by a PLL is modelled taking full account of the delay, providing the first report of the location of all the infinity of poles of the controlled oscillator system function. This provides a well-characterised basic subsystem from which more complex architectures may be composed, either as nested control loops or as coupled oscillators. The theoretical considerations are supported by experimental observations. The first observation is reported of an OEO exhibiting giant phase modulated oscillation analogous to the FM mode regime of an actively mode-locked laser^15^. The giant phase modulation phenomenon is the source of a modulation instability of a PLL-OEO system which is described by the analysis and observed experimentally. Nevertheless, the analysis and experimental observations presented demonstrate that stable phase lock operation and optimum phase noise performance is achievable if the multimode operation of the basic oscillator is accounted for in the phase lock analysis.

### Free optoelectronic oscillator system poles

**Figure 1:**
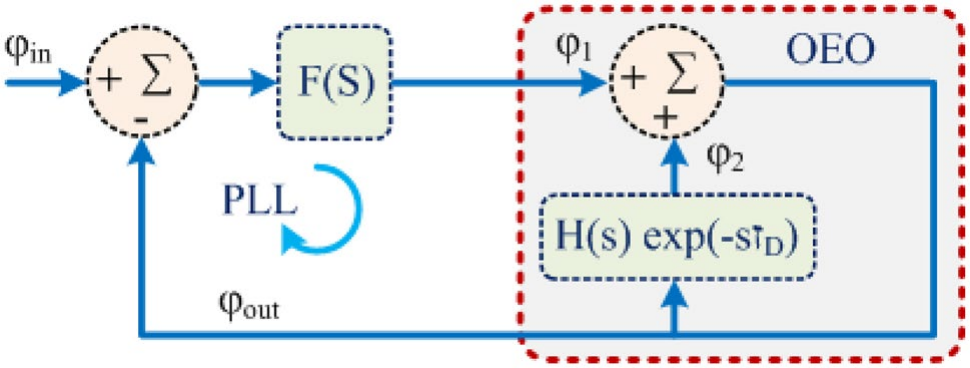
Leeson phase model of a phase locked optoelectronic oscillator system. The phase locked loop forms a negative feedback loop composed of the phase sensitive detector (PSD), loop filter, and optoelectronic oscillator (OEO). The PSD is depicted by the signed summation block on the left that measures the phase error $$ \phi_{in} - \phi_{out}$$. The loop filter is depicted by a block labelled by its system function $$ F$$. The OEO is depicted by the contents of the red dashed box that form a positive feedback loop. The rectangle represents the combined action in the Laplace transform domain of the fibre optic delay line and RF bandpass filter, where $$ \tau_{D}$$ is the time delay and $$H$$ is the baseband equivalent system function of the RF bandpass filter. The summation block represents a phase shifter that introduces a single pass phase shift equal to the tuning control $$\phi_{1}$$.

Consider the controlled optoelectronic oscillator shown within the dashed box in Fig. 1. The system function $G$ of a Leeson model^16^ of the optoelectronic oscillator is given by:

1 $$ \begin{array}{*{20}c} {G\left( s \right) = \frac{1}{{1 - \frac{1}{{1 + \tau_{R} s}}\exp \left( { - \tau_{D} s} \right)}}} \\ \end{array} $$

where ${ }\tau_{D}$ is the delay and $\tau_{R}$ is the on-resonance group delay of the RF bandpass filter that promotes single-mode oscillation. For simplicity, a single-pole baseband-equivalent model of the bandpass filter is used (see supplementary material).

The poles of $G$ are located at the roots of the equation:

2 $$ 1 + s\tau_{R} - \exp \left( { - s\tau_{D} } \right) = 0 $$

which may be cast into the same form:

3 $$ \begin{array}{*{20}c} {\left( {\rho + \tau_{D} s} \right)\exp \left( {\rho + \tau_{D} s} \right) = \rho \exp \left( \rho \right)} & ; & {\rho = \tau_{D} /\tau_{R} } \\ \end{array} $$

as the definition of the Lambert $w$ function^17^:

4 $$ w\left( z \right)\exp \left( {w\left( z \right)} \right) = z $$

Consequently, the roots $s_{k}$ are given by:

5 $$ \begin{array}{*{20}c} {s_{k} \tau_{D} = { }w_{k} \left( {\rho \exp \left( \rho \right)} \right) - { }\rho } & ; & {k \in {\mathbb{Z}}} \\ \end{array} $$

where the integer $$k$$ indexes the countable infinity of branches of the multivalued Lambert $$w$$ function. The Lambert $$ w$$ function organises its solutions by increasing negative real part with increasing magnitude of the branch index Conveniently, the Lambert $$w$$ function is a MATLAB supplied function.

Expressed in terms of the real $\sigma$ and imaginary $i\omega { }$ parts of the Laplace transform variable $s = \sigma + i\omega$, Eq. (2) separates into two coupled real equations:

6 $$ \tau_{D} \sigma = - \frac{1}{2}\ln \left( {\left( {1 + \tau_{R} \sigma } \right)^{2} + \left( {\tau_{R} \omega } \right)^{2} } \right) $$

7 $$ \begin{array}{*{20}c} {\begin{array}{*{20}c} {\begin{array}{*{20}c} {\tau_{D} \omega = 2\pi k - \tan^{ - 1} \left( {\tau_{R} \omega /\left( {1 + \tau_{R} \sigma } \right)} \right)} & ; & {k \in {\mathbb{Z}}} \\ \end{array} } \\ \end{array} } \\ \end{array} $$

Equation (6) defines an implicit curve in the complex plane parameterised by $\tau_{R} \omega$ , referred to herein as the pole locus, on which the poles must lie. The bounded range of the arctangent in Eq. (7) localises each pole to a neighbourhood of $\tau_{D} \omega = 2\pi k$. Equation (6) & (7) may be iterated to find the precise locations of the poles as an alternative to evaluating the Lambert $w$ function.

For large delay $\tau_{D} \gg \tau_{R}$, Eq. (6) & (7) simplify to:

8 $$ \tau_{D} \sigma = - \frac{1}{2}\ln \left( {1 + \left( {\tau_{R} \omega } \right)^{2} } \right) $$

9 $$ \begin{array}{*{20}c} {\begin{array}{*{20}c} {\begin{array}{*{20}c} {\tau_{D} \omega = 2\pi k - \tan^{ - 1} \left( {\tau_{R} \omega } \right)} & ; & {k \in {\mathbb{Z}}} \\ \end{array} } \\ \end{array} } \\ \end{array} $$

**Figure 2:**
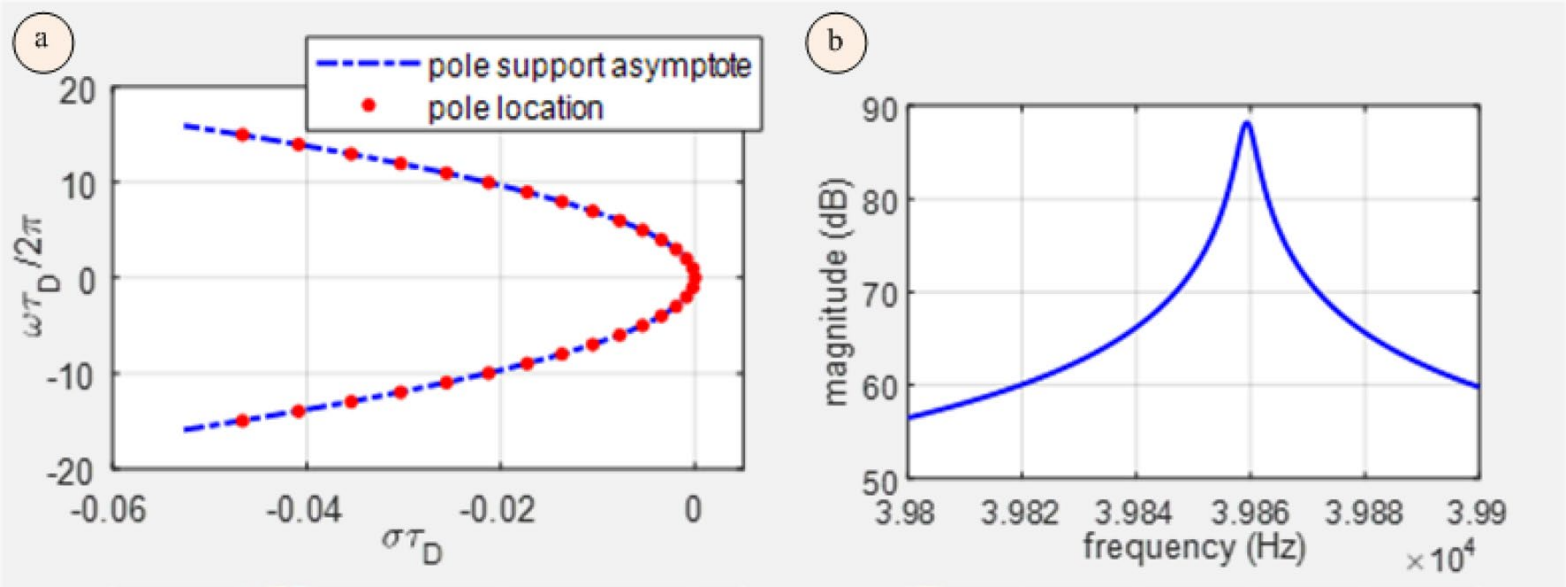
(**a**) A comparison of the poles of the system function of a free time delay oscillator and the asymptotic curve defined by Eq. (8); (**b**) Bode plot of the transfer function of an optoelectronic voltage-controlled oscillator relative to an orthodox voltage-controlled oscillator with equivalent static tuning sensitivity-in a neighborhood of the first sidemode resonance.

The explicit pole locus defined by Eq. (8) provides an excellent fit to the exact distribution of the poles for representative values of the $\tau_{D} , \tau_{R}$ parameters (see Fig. 2a) and provides an upper bound to their real part. Such curves were introduced by Yanuck^18^ using the term ‘pseudo-continuous spectrum’ and are of utility in establishing sufficient conditions for the stability of controlled time delay oscillator systems without explicit solution in terms of elementary or special functions.

### Giant phase modulation

While the system function of the optoelectronic oscillator shares with an orthodox voltage-controlled oscillator a pole at the origin representing a perfect integrator, it possesses in addition, a countable infinity of complex conjugate pairs of poles in the left-hand half-plane close to the imaginary axis representing sidemode resonances. Referring to Fig. 1 and Eq. (1), tuning phase modulation:

10 $$ \phi_{1} = {\Delta }\phi \cos \left( {\omega t} \right) $$

with amplitude ${\Delta }\phi$ and frequency $\omega$ causes oscillation phase modulation:

11 $$ \begin{array}{*{20}c} {\phi_{out} = \mu \cos \left( {\omega t + \xi } \right)} \\ \end{array} $$

where:

12 $$ \begin{array}{*{20}c} {\begin{array}{*{20}c} {\mu = \left| {G\left( {i\omega } \right)} \right|\Delta \varphi } & ; & {\xi = \arg \left( {G\left( {i\omega } \right)} \right)} \\ \end{array} } \\ \end{array} $$

Under conditions where all significant sidebands of the modulated oscillation fall well within the passband of the RF-bandpass filter, the complex envelope is given by:

13 $$ \begin{array}{*{20}c} {u\left( t \right) = \exp \left[ {i\left( {{\upomega }_{0} t + \mu \cos \left( {\omega t + \xi } \right)} \right)} \right] = \exp \left( {i{\upomega }_{0} t} \right)\mathop \sum \limits_{n = - \infty ,\infty } i^{n} J_{n} \left( \mu \right)\exp \left( {in\left( {\omega t + \xi } \right)} \right)} \\ \end{array} $$

where ${\upomega }_{0}$ is the natural frequency of the unmodulated oscillation. The right-hand side of Eq. (13) follows from the Jacobi-Anger expansion. The modulated oscillation is known as a Bessel super mode and has a comb spectrum with spectral lines at intervals of the modulation frequency.

The on-sidemode-resonance phase modulation gain $G\left( {i\omega_{k} } \right)$ may be estimated as:

14 $$ G\left( {i\omega_{k} } \right)\sim \frac{1}{{2\pi^{2} k^{2} }}\left( {\frac{{\tau_{D} }}{{\tau_{R} }}} \right)^{2} $$

which for representative parameter values $k = 1,$ $\tau_{R} \sim 86 ns, \tau_{D} \sim 25 \mu s$, evaluates to 4281 ($73 dB$). The phase modulation gain at the same frequency of an orthodox VCO with the same tuning sensitivity is:

15 $$ G\left( {i\omega_{k} } \right) = \frac{1}{2\pi k} $$

which evaluates to 0.1591 (-16 dB). The 89 dB greater gain at the first sidemode resonance of the OEO compared to the VCO is confirmed by the plot in Fig. 2b. Equation (14) is only valid for $\tau_{D} \gg$ $\tau_{R}$. However, Eq. (1)) and the theoretical description of tuning phase modulation remains valid as $\tau_{D} \to 0$ but the character of the oscillator progressively evolves from a multimode time delay oscillator with properties dominated by the delay line to a classical single mode oscillator with properties dominated by the RF bandpass filter (see supplementary material).

**Figure 3:**
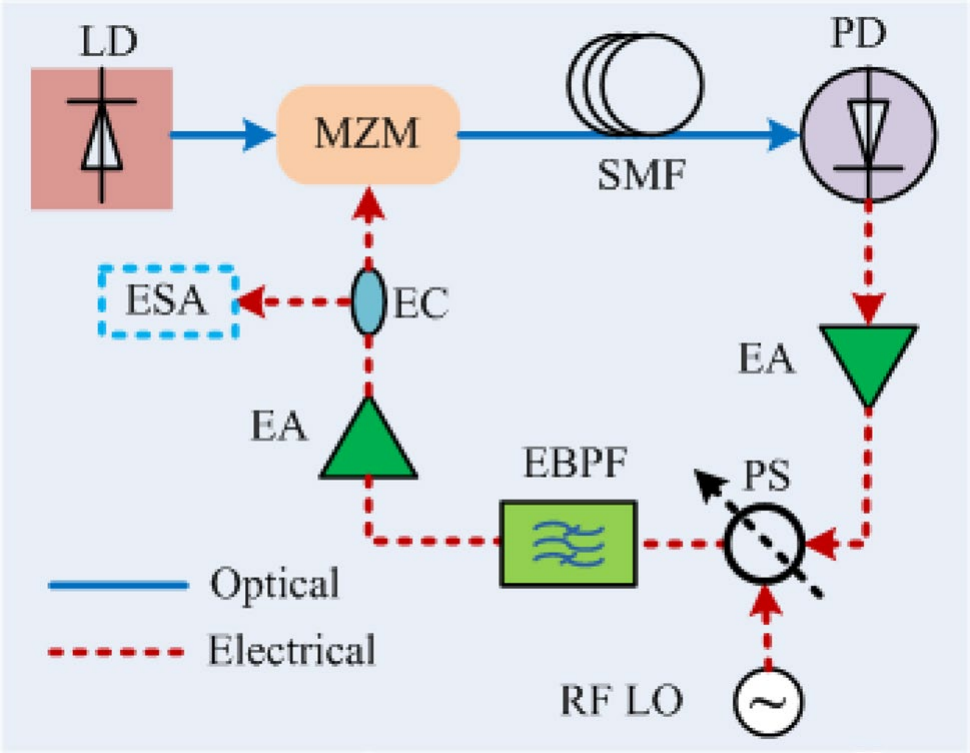
Schematic diagram of the experimental setup for the observation of giant phase modulation. *LD* Laser diode, *MZM* Mach–Zehnder modulator, *SMF* Single mode fiber, *PD* Photodiode, *EA* Electrical amplifier, *PS* Phase shifter, *EBPF* Electrical bandpass filter, *EC* Electrical coupler, *ESA* Electrical spectrum analyzer, *RF LO* Radio frequency local oscillator.

To confirm these phase modulation resonances experimentally, a small voltage modulation is applied to a voltage-controlled RF phase shifter inserted within the OEO loop. The experimental arrangement is shown schematically in Fig. 3. An Analog Devices HMC931 is used as the voltage-controlled phase shifter (PS). A custom 10 GHz resonator having quality factor of around ∿2700 is used as the electrical bandpass filter (EBPF). A distributed feedback (DFB) laser having an output power of 80 mW (Em650 from G & H) with an operating wavelength near ∿1550.12 nm is used as the optical source. The electrical amplifiers are off-the-shelf RF power amplifiers having a gain of $$ 16 dB$$. Three similar electrical amplifiers were used in the loop to compensate the extra insertion losses caused by the EBPF $$\left( { \sim 10 dB} \right)$$ and the PS $$\left( { \sim 4 dB} \right)$$.

**Figure 4:**
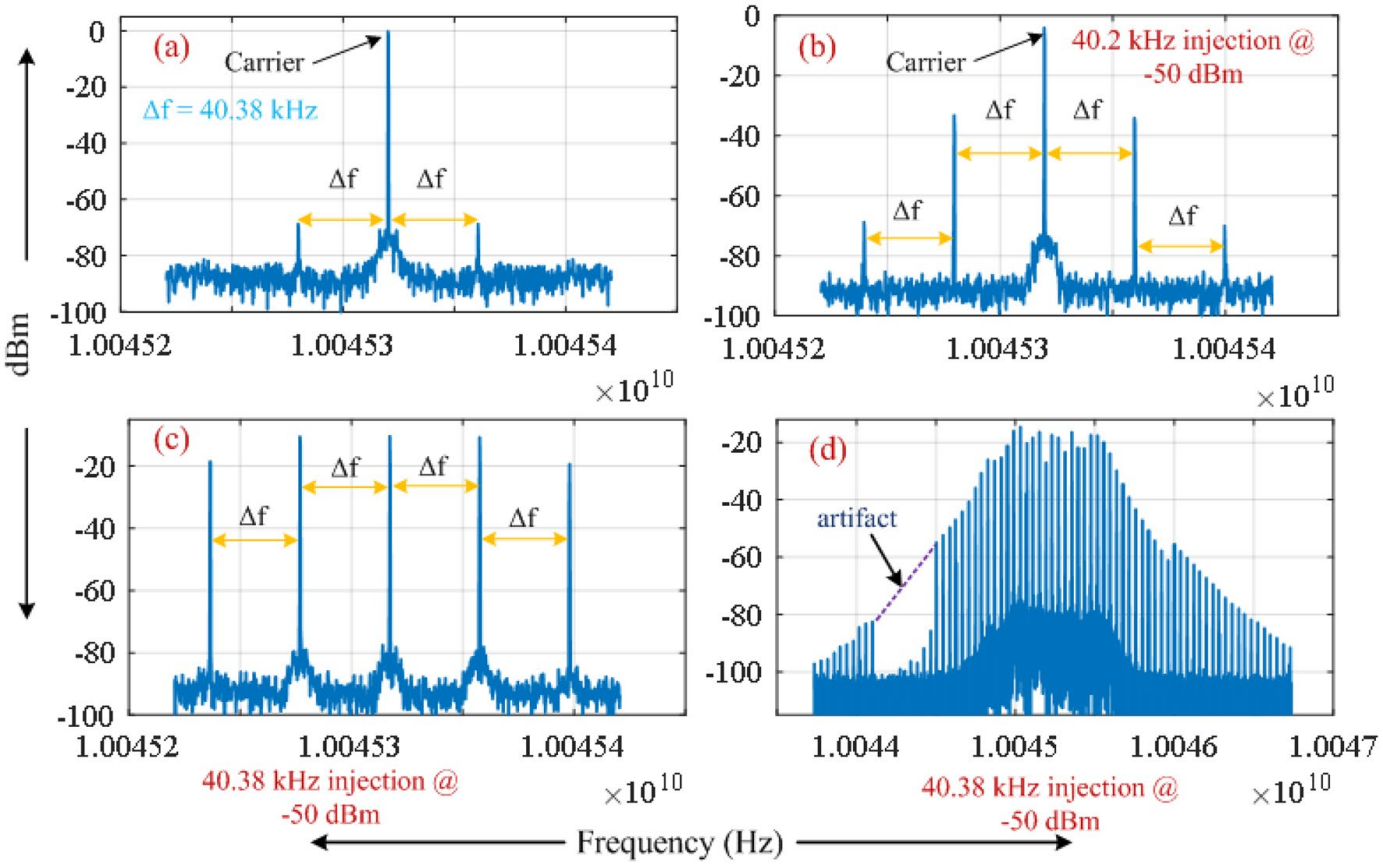
Measured electrical spectrum for (**a**) the free OEO and (**b**–**d**) with small (− 50 dBm) injection of sinusoidal intra-loop phase modulation via the phase shifter having frequencies close to the inter mode frequency interval. A resolution bandwidth of 100 Hz is used for all the measurements. A span of 200 kHz is used for (**a**–**c**); whereas a span of 3 MHz is used for (**d**). The artifact in (**d**) is a text file error when saving a spectrum with a large number of points.

Figure 4a shows the measured spectrum of the RF output of the OEO in the absence of applied modulation. The sidemode resonances excited by residual intra-loop phase fluctuations are separated from the oscillation frequency by the reciprocal of the delay (~ 25 $\mu s$) produced by a 5 km optical fibre coil. A sinusoidal voltage with 40 kHz frequency and $- 50 dBm$ power is applied to the phase shifter (PS) to inject intra-loop phase modulation. Figure 4b–d shows the resulting measured electrical spectrum of the RF output of the OEO. The RF spectrum of the free OEO presented in Fig. 4a does not change when the injected frequency is 40 kHz. However, at the modulation frequency ∼40.20 kHz the sidemodes within the RF spectrum gain considerable power with injected phase modulation (Fig. 4b) in comparison to the spectrum without any injected phase modulation (Fig. 4a). At modulation frequencies close to resonance (∿40.38 kHz), the outcome is giant phase modulation of the carrier confirming the prediction of Eq. (14). Consistent with Eq. (13), the giant phase modulation generates a comb spectrum with peaks of similar magnitude (Fig. 4c) at intervals of the modulation frequency extending over a broad band (Fig. 4d) comparable to the 3.7 MHz $- 3 dB$ bandwidth of the EBPF.

The near-singular sidemode resonances in the phase modulation transfer function of the optoelectronic oscillator are responsible for the phenomenon of giant phase modulation. When an OEO is placed within a phase control loop the sidemode resonances are a source of modulational instability. Spontaneous giant phase modulation is almost certain to be observed if the controller design does not take account of the sidemode resonances.

### Controlled optoelectronic oscillator system poles

The behaviour of a phase locked loop (PLL) is accurately captured by a dynamical system model with the phase of the voltage-controlled oscillator (VCO) as state variable. An orthodox VCO is modelled as a perfect integrator characterized by its tuning sensitivity $K_{VCO}$ ($Hz/V$). The phase sensitive detector (PSD) may be based on a balanced mixer or other approximation to a four-quadrant multiplier, which provides an approximately sinusoidal response with $2\pi$ period. An alternative implementation based on a sequential phase detector with charge pump provides a linear response over an interval of $\left( { - 2\pi ,2\pi } \right)$ outside of which the response saturates. In either case, the PSD is characterised by its sensitivity $K_{PSD}$ ($V/rad$) to small phase differences. The loop filter is modelled by a linear system of differential equations, which is expressed equivalently but more conveniently by the system function in the Laplace transform domain. Indeed, subject to linearization, the closed loop system function of the complete PLL-VCO may be derived and its poles (and zeros) located. Since the number of poles is small, it is a relatively simple design matter to place all the poles in the left-hand half-plane of the Laplace transform domain to ensure stability.

On the other hand, an OEO may be tuned by inserting a voltage-controlled phase shifter within the oscillating path (see Fig. 3). The path contains an optical fibre coil of substantial length $\left( {\sim 5km} \right)$ and hence long delay $(\sim 25 \mu s$). The OEO oscillation essentially accumulates a phase step on each round trip resulting in a staircase approximation of the phase ramp provided by an orthodox VCO. The passband of the RF filter is large (~ 3.7 MHz) compared to the frequency interval between adjacent sidemodes ($\sim 40 kHz$) and hundreds of roundtrips are necessary before the smoothing of the staircase it provides is effective. The OEO is an example of a time delay oscillator and its behaviour as a voltage-controlled oscillator differs substantially from an orthodox VCO. Systems with time delays such an OEO are challenging to control^19^. Their stability analysis is complicated by a system function possessing an infinity of poles. Consequently, while a controller may be designed to place a finite number of known poles in the lefthand half-plane of the Laplace transform domain, one rarely can be certain that the same controller has not moved one or more of the remaining infinity of poles into the righthand half-plane, thereby leading to instability.

It is convenient to take the signal input to the phase sensitive detector from the output coupler of the oscillator, which leads to the configuration illustrated schematically by Fig. 1. The complete system function is:

16 $$ K\left( s \right) = \frac{F\left( s \right)}{{1 + F\left( s \right) - \frac{1}{{1 + \tau_{R} s}}\exp \left( { - \tau_{D} s} \right)}} $$

where $F{ }$ is the controller system function. The poles of $K$ are located at the roots of the equation:

17 $$ 1 + F\left( s \right) - \frac{1}{{1 + \tau_{R} s}}\exp \left( { - s\tau_{D} } \right) = 0 $$

Equation (17) separates into two coupled real equations:

18 $$ \tau_{D} \sigma = - \frac{1}{2}\ln \left( {\left( {1 + \tau_{R} \sigma } \right)^{2} + \left( {\tau_{R} \omega } \right)^{2} } \right) - \frac{1}{2}\ln \left( {\left| {1 + F\left( s \right)} \right|^{2} } \right) $$

19 $$ \begin{array}{*{20}c} {\tau_{D} \omega = 2\pi k - \tan^{ - 1} \left( {\tau_{R} \omega /\left( {1 + \tau_{R} \sigma } \right)} \right) - \arg \left( {1 + F\left( s \right)} \right)} & ; & {k \in {\mathbb{Z}}} \\ \end{array} $$

that differ from Eq. (6) & Eq. (7) by the addition of the final term on the righthand sides.

In the case of a proportional integral controller:

20 $$ F\left( s \right) = \kappa + \frac{1}{{s\tau_{I} }} \equiv \kappa \frac{{1 + s\tau_{1} }}{{s\tau_{1} }} $$

where $\kappa$ is the proportional gain, $\tau_{I}$ is a time constant that characterises the integrator, and $\tau_{1} = \kappa \tau_{I}$ is a time constant that characterises an equivalent lead-lag network.

For representative parameter values ($\tau_{R} \sim 86 ns, \tau_{D} \sim 25 \mu s, \tau_{I} \sim 100ms)$ the time constants $\tau_{R} \ll \tau_{D} \ll \tau_{I}$ are roughly evenly distributed over three orders of magnitude. Consequently, the standard assumption that the oscillator behaves as an ideal integrator may be used to place the inner poles. Specifically, the approximation:

21 $$ 1 - \begin{array}{*{20}c} {\frac{1}{{1 + \tau_{R} s}}\begin{array}{*{20}c} {\exp \left( { - \tau_{D} s} \right)\sim s\tau_{D} } \\ \end{array} } & ; & {\left| {s\tau_{R} } \right| \ll \left| {s\tau_{D} } \right| \ll 1} \\ \end{array} $$

is made to reduce Eq. (17) to:

22 $$ \begin{array}{*{20}c} {F\left( s \right) + s\tau_{D} = 0} & ; & {\left| {s\tau_{D} } \right| \ll 1} \\ \end{array} $$

Substitution of Eq. (20) results in a quadratic equation describing damped simple harmonic motion:

23 $$ 1 + 2\xi \left( {\tau_{F} s} \right)^{2} + \left( {\tau_{F} s} \right)^{2} = 0 $$

where:

24 $$ \tau_{F} = \sqrt {\tau_{I} \tau_{D} } $$

is the period of the natural oscillation and:

25 $$ \xi = \frac{1}{2}\kappa \sqrt {\frac{{\tau_{I} }}{{\tau_{D} }}} $$

is the damping factor. The two roots:

26 $$ \tau_{F} s = - \xi \pm \sqrt {\xi^{2} - 1} $$

provide an accurate estimate of the location of the two inner poles of $K$. Over damping corresponds to $\xi > 1$ and a reciprocal pair of real poles. Critical damping corresponds to $\xi = 1$ and a double pole on the real axis. Under damping corresponds to $\xi < 1$ and a complex conjugate pair of poles situated in the left-hand half-plane on a circle of radius $1/\tau_{F}$ centred on the origin. The choice $\xi = 1/\sqrt 2$ provides responsive dynamics. The natural frequency is best set close to the frequency (~ 100 Hz) at which the system reference phase noise crosses over the phase noise of the free oscillator.

Beyond the inner pole region $$\begin{array}{*{20}c} {F\left( s \right) \to \kappa } \\ \end{array}$$ and Eq. (18) approaches:

27 $$ \begin{array}{*{20}c} {\begin{array}{*{20}c} {\tau_{D} \sigma = - \frac{1}{2}\ln \left( {\left( {1 + \tau_{R} \sigma } \right)^{2} + \left( {\tau_{R} \omega } \right)^{2} } \right) - \ln \left( {1 + \kappa } \right)} \\ {\begin{array}{*{20}c} { \approx - \frac{1}{2}\ln \left( {1 + \left( {\tau_{R} \omega } \right)^{2} } \right) - \ln \left( {1 + \kappa } \right)} & ; & {\tau_{R} \ll } \\ \end{array} \tau_{D} } \\ \end{array} } \\ \end{array} $$

The outer poles approach the pole locus of a proportionally controlled oscillator (see supplementary material Fig. 1A and associated discussions). The two controller parameters available to the designer have been used to place the inner poles, it is good fortune that the outer poles are in the left-hand half-plane ensuring stability.

In the example given, it is significant that $\arg \left( F \right) \to 0$ outside the inner pole region. The phase of $F$ plays a critical role in Eq. (18) via the term:

28 $$ \begin{array}{*{20}c} {\begin{array}{*{20}c} { - \frac{1}{2}\ln \left( {\left| {1 + F\left( s \right)} \right|^{2} } \right) = - \frac{1}{2}\ln \left( {1 + \left| F \right|^{2} + 2\left| F \right|\cos \left( {\arg \left( F \right)} \right)} \right)} \\ \end{array} } \\ \end{array} \sim - \left| F \right|\cos \left( {\arg \left( F \right)} \right) $$

**Figure 5:**
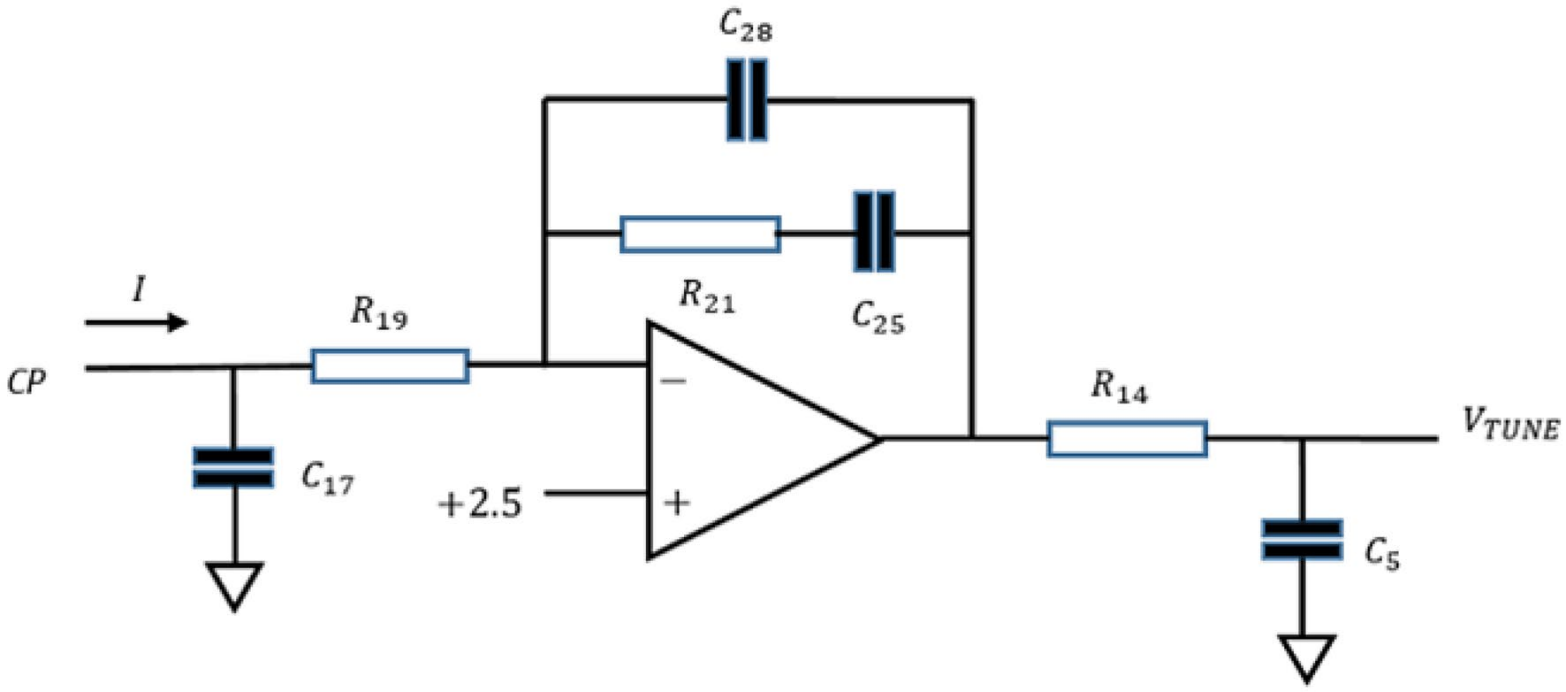
The active loop filter circuit included on the phase locked loop evaluation board used for experiments. R19, R21, R14, C17, C28, C25 and C5 are labeled verbatim from the HMC703 evaluation board. The 2.5 V is a bias used to permit a single ended power supply but otherwise acts as a signal ground.

Stability is only guaranteed if the cosine is positive. The proportional integral controller retains proportional control at high frequencies, yet prima facie, it is desirable that the controller relinquishes control completely at frequencies where the free oscillator provides superior phase noise performance. In practice additional poles are inserted into the loop filter transfer function to provide design freedom for a variety of purposes such as phase detector comparison frequency rejection, improved tracking of ramped or chirped reference sources and phase noise spectral shaping. The PLL evaluation board active loop filter (Fig. 5) used in experiments introduces three such additional poles.

A single pole low-pass filter contributes a phase shift that accumulates to $- \pi /2$ over a range of frequencies spanning roughly a decade either side of its breakpoint frequency. Consequently, a section of the pole locus can enter the righthand half-plane and stability is no longer guaranteed. If that section includes a sidemode resonance a modulational instability would be guaranteed if not for the small gain margin provided by the RF bandpass filter. A three-pole low pass filter designed without regard to the OEO sidemode may shift an outer pole with relative ease into the left-hand half-space of the Laplace transform domain (see Fig. 2A supplementary material).

### Loop filter design and experimental results

**Figure 6:**
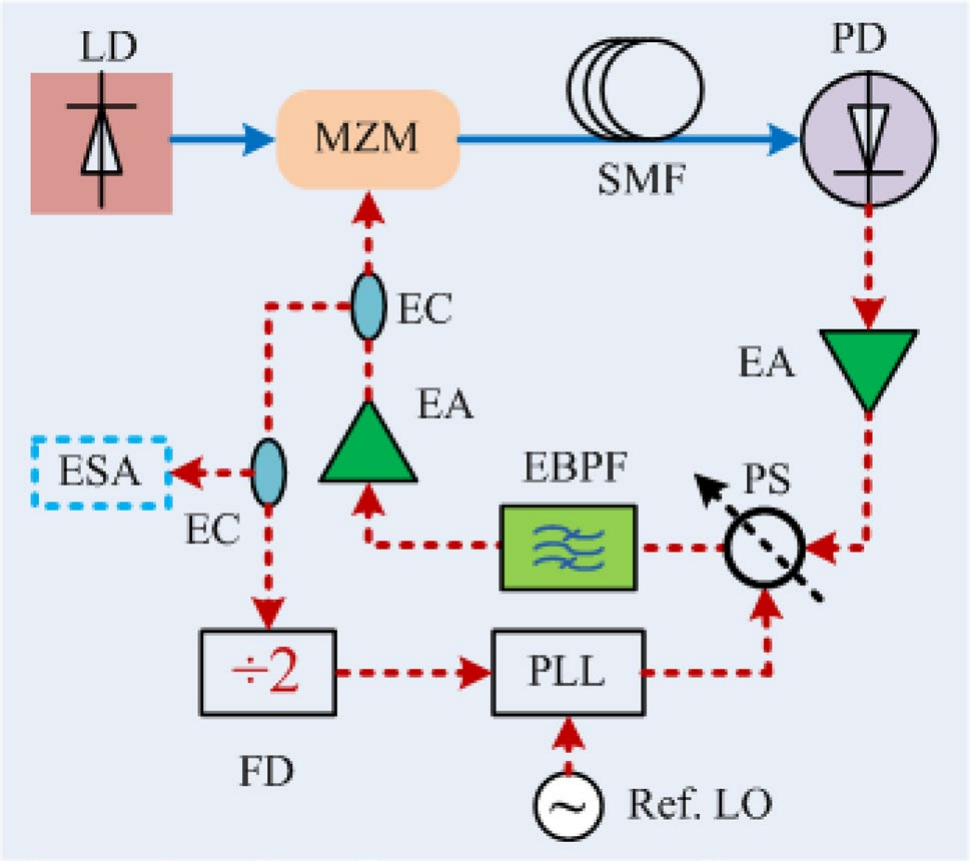
Schematic diagram of the experimental setup for phase locking the OEO to the reference *LO PLL* Phase lock loop; *FD* Frequency divide, *SSA* Signal source analyzer; Ref. *LO* Reference local oscillator.

Off-the-shelf components are used to demonstrate the phase locking of a single loop optoelectronic oscillator. Figure 6 shows a schematic diagram of the experimental arrangement. An Analog Devices HMC703 is used as the PLL in this experiment. A Keysight N5166B CXG RF vector signal generator is used to provide a 100 MHz carrier with power of 6 dBm as reference input. The OEO operates at 10 GHz which is above the 8 GHz input frequency limit of the HMC703 consequently a $\div 2$ frequency divider is used before the PLL to bring the signal frequency into the operating range of the PLL. A Keysight signal source analyzer (SSA) E5052B is used to measure the phase noise spectrum of the PLL-OEO system.

**Figure 7:**
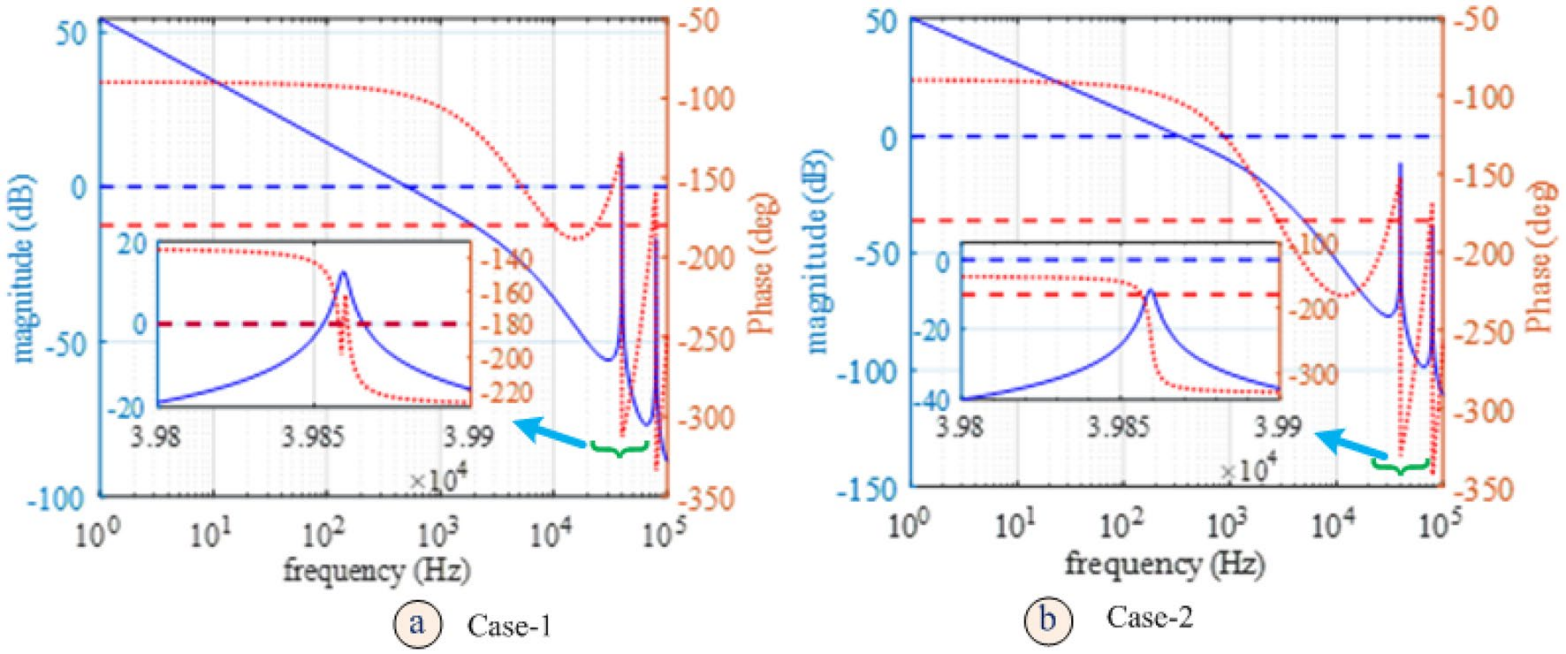
Open loop Bode plot for the PLL-OEO for the two different loop filter designs considered. The inset shows the first side-mode resonance for two different sets of loop filter component values listed in Table 1.

The PLL evaluation board active loop filter (Fig. 5) used for experiments provides a controller system function:

29 $$ F\left( s \right) = \kappa \frac{{\tau_{1} }}{{\tau_{2} }}.\frac{{1 + s\tau_{2} }}{{s\tau_{1} }}.\frac{1}{{1 + s\tau_{3} }}.\frac{1}{{1 + s\tau_{4} }}.\frac{1}{{1 + s\tau_{5} }} $$

which, in addition to the lead-lag term, introduces three single pole factors. The time constants $$\tau_{1} , \tau_{2} , \tau_{3} , \tau_{4} , \tau_{5}$$ are defined by the active filter circuit component values and $$\kappa$$ is the proportional gain. Representative values of the parameters $$\kappa$$, $$\tau_{1} , \tau_{2} , \tau_{3} , \tau_{4} , \tau_{5}$$ are given in Table 1 evaluated for two example component sets (Case-1 and Case-2) (see supplementary material). The component values listed were provided by filter design software with a charge pump current setting of $$I_{CP} = 2.5 mA$$ and a static voltage-controlled oscillator gain setting of $$K_{VCO} = 4 kHz/V$$ as measured experimentally. The filter design software selects component values with no regard to sidemode resonances. The optoelectronic oscillator operated at 10 GHz. The system reference was provided by a 100 MHz RF source. Consequently, the PLL used a $$N = 100$$ frequency divider, which has the effect of reducing the loop gain by the same factor. Both sets of component values provided a stable PLL-VCO (not OEO) with substantial phase and gain margins $$ \sim 80^\circ \& 25 dB$$. On the other hand, the Bode plot for the PLL-OEO for the two component sets over a range of offset frequencies up to $$100 kHz$$ is given in Fig. 7. Superficially the Bode plots indicate stable operation with similar phase and gain margins until one inspects a small neighborhood of the first sidemode resonance at $$\sim 40 kHz $$ shown in the inset of Fig. 7 where the phase lag passes through $$- 180^\circ$$. For Case-1 parameter values, the loop gain exceeds unity by up to $$\sim 10 dB$$ ensuring instability. The Case-2 parameter values reduces the loop gain at the first side-mode resonance below $$\sim - 9 dB$$ suppressing the instability. For the first component set spontaneous giant phase modulated oscillation (modulational instability) is observed experimentally for a charge pump current of $$800 \mu A$$ or greater. The growth of the phase modulation is limited only by saturation within the control loop and can be up to $$\pm 2N\pi \sim \pm 630 rads$$. For Case 2 parameter values modulation instability is not observed experimentally for charge pump current up to $$ 2.5 mA$$. The respective predicted outer pole locations of the complete system are illustrated by supplementary material Fig. 2A.

**Table 1:** Loop filter component and system parameter values; (a) Component values for two different instances of loop filter design; (b) model parameters derived from component values (see supplementary material for formulae).

(a)										
Case	${R}_{19}$ $(k\Omega )$	${R}_{21}$ $(k\Omega )$	${R}_{14}$ $(k\Omega )$	${C}_{17}$ $(nF)$	${C}_{25}$ $(nF)$	${C}_{28}$ $(nF)$	${C}_{5}$ $(nF)$	${K}_{VCO}$ $(kHz/V)$	*N*	${I}_{CP}$ $(mA)$
1	33	33	1.8	0.47	47	$1$	$4.7$	$4$	100	2.5
2	22	22	2.2	2.2	220	$3.3$	$4.7$	$4$	100	2.5

(b)										
Case	$\kappa$	${\tau }_{1}$ $(\mu s)$	${\tau }_{2}$ $(\mu s)$	${\tau }_{3}$ $(\mu s)$	${\tau }_{4}$ $(\mu s)$	${\tau }_{5}$ $(\mu s)$	${\tau }_{D}$ $(\mu s)$	${\tau }_{R}$ $(ns)$		
1	0.081	1584	1551	32.31	15.51	8.46	25	86		
2	0.054	4913	4840	71.53	48.40	10.34	25	86

The loop-filter design freedom that is required to achieve performance objectives other than stability such an engineered phase noise spectrum is seriously curtailed by modulation instability. The restoration of design freedom requires methods of suppressing the spurious resonances that do not re-introduce system reference phase noise. The possibilities include self-injection / self-phase locking, which involve more than one loop, and/or low-noise methods of reducing RF filter bandwidth, which is a challenge.

**Figure 8:**
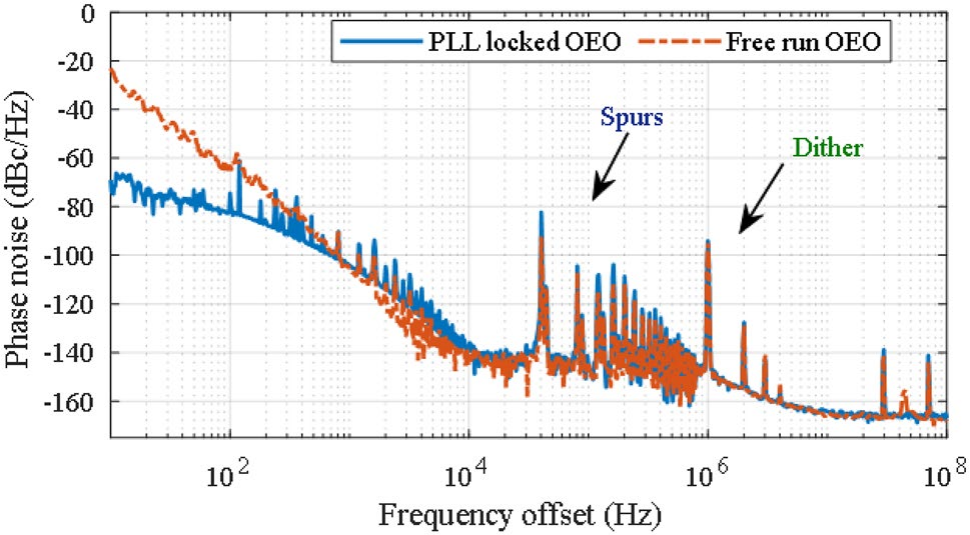
Measured phase noise of the free OEO and phase locked OEO. 100 correlation is used for the measurement.

Figure 8 shows the measured phase noise of the OEO without (free OEO) and with PLL locking (PLL locked OEO). 100 correlations are used for the measurement. A phase noise of − 141 dBc/Hz @ 10 kHz offset is attained for a carrier frequency of 10.045 GHz. With an increase in the number of correlations to 1000, the measured phase noise of the OEO improved to − 145 dBc/Hz @ 10 kHz offset, however the measurement time thereby becomes prolonged. The spurious spectral lines at 1 MHz and harmonics are caused by the laser frequency dither which is applied to mitigate phase noise due to double Rayleigh scattering (DRS)^20^. The phase locked spectrum also shows that the bandwidth of the active loop filter needs to be reduced further to optimise the crossover from the locked OEO to the free OEO phase noise. If required, the frequency drift compensation can be enlarged further using the method presented in^21^.

## Conclusion

This paper reports the findings of a study of an optoelectronic oscillator (OEO) with large delay under proportional and proportional integral control by a phase-locked loop (PLL). The study is the first to fully account for the OEO delay in responding to a tuning stimulus including the location of all the countable infinity of poles of the system function. This provides a well characterised basic subsystem from which more complex architectures may be composed, either as nested control loops or as coupled oscillators. The theoretical considerations are supported by experimental observations. The first observation is reported of an OEO exhibiting giant phase modulated oscillation that is related to the FM-mode regime of an actively mode-locked laser but without the pulsed mode regime. Giant phase modulation is the source and spontaneous giant phase modulation (i.e., regenerative FM-mode regime mode-locking) is a manifestation of a modulation instability of a PLL-OEO system which is described by the analysis and observed experimentally. Nevertheless, the analysis and experimental observations, including a prototype 10 GHz PLL-OEO phase noise spectral density achieving $$ - 80dBc/Hz $$ at $${\text{at}} 10 Hz$$ and $$- 145dBc/Hz $$ at $$10 kHz$$ demonstrate that stable phase lock operation and near optimum phase noise performance is achievable provided full account of the multimode character of the OEO is taken in the phase-lock analysis.

## Supplementary Information

Supplementary Information.

## Data Availability

The datasets used and/or analysed during the current study available from the corresponding author on reasonable request.
