# Supplementary material for: On the stability & phase locking to a system reference of an optoelectronic oscillator with large delay
Source: Sci Rep. 2023 Mar 14;13:4207. doi: 10.1038/s41598-023-31248-w (PMC10014935; doi:10.1038/s41598-023-31248-w)
Supplement: Supplementary file 1 — Supplementary Information. [file 41598_2023_31248_MOESM1_ESM.docx]

**On the stability & phase locking to a system reference of an optoelectronic oscillator with large delay**

Mehedi Hasan^1*^, Charles Nicholls^2^, Trevor Hall^1^

^1^ Photonic Technology LAB, University of Ottawa, 25 Templeton Street, Ottawa, ON, Canada, K1N 6X1

^2^ NANOWAVE Technologies Inc., 6 Gurdwara Rd, Nepean, ON, Canada, K2E 8A3

*To whom correspondence should be addressed; E-mail: [mhasa067@uottawa.ca](mailto:mhasa067@uottawa.ca)

**Supplementary material**

**Baseband equivalent transfer function of a bandpass filter**

Let the bandpass filter be described by the convolution:

$y=f\otimes x$ (A1)

where $f$ is the real impulse response of the filter;

$x\left( t \right)= u\left( t \right)\exp\left( i\omega_{0}t \right)$ (A2)

$\begin{matrix} \begin{matrix} y\left( t \right)= v\left( t \right)\exp\left( i\omega_{0}t \right) \end{matrix} \end{matrix}$ (A3)

$u$, $v$ are the respective complex envelopes of $x$, $y$ and $\omega_{0}$ is the nominal carrier frequency:

Introducing the Fourier transform pairs:

$x,y,f,u,v⟷X,Y,F,U,V$ (A4)

it follows that:

$Y\left( \omega\right)=F\left( \omega\right)X\left( \omega\right)$ (A5)

$X\left( \omega_{0}+\omega\right)=U\left( \omega\right)$ (A6)

$Y\left( \omega_{0}+\omega\right)=V\left( \omega\right)$   (A7)

Hence:

$V\left( \omega\right)=Y\left( \omega_{0}+\omega\right)=F\left( \omega_{0}+\omega\right)X\left( \omega_{0}+\omega\right)=H\left( \omega\right) U\left( \omega\right)$ (A8)

where:

$H\left( \omega\right)= F\left( \omega_{0}+\omega\right)$  (A9)

is the baseband equivalent transfer function of the bandpass filter.

The transfer function of a two-pole bandpass filter is described by:

$F\left( \omega\right)=\frac{1}{1+iQ\left( 1+\frac{\omega-\omega_{0}}{\omega_{0}}-\frac{1}{1+\frac{\omega-\omega_{0}}{\omega_{0}}} \right)}$ (A10)

where $Q={\omega_{0}}/{\Delta\omega}$ is the quality factor, $\omega_{0}$ is the passband centre frequency, and $\Delta\omega$ is the $-3dB$ bandwidth.

Consequently, the baseband equivalent transfer function is given by:

$H\left( \omega\right)=\frac{1}{1+iQ\left( 1+\frac{\omega}{\omega_{0}}-\frac{1}{1+\frac{\omega}{\omega_{0}}} \right)}$ (A11)

Noting the offset frequencies $\omega$ of interest are small $\left| \omega/{\omega_{0}} \right|\sim{10}^{-6}-{10}^{-4}$ one may apply the binomial expansion to first order:

$\frac{1}{1+\frac{\omega}{\omega_{0}}}=1-\frac{\omega}{\omega_{0}}+\ldots$  (A12)

to yield a highly accurate single pole baseband equivalent transfer function:

$\begin{matrix} H\left( \omega\right)\approx\frac{1}{1+i\omega\tau_{R}} & ; & \tau_{R}=\frac{2Q}{\omega_{0}}=\frac{1}{{\Delta\omega}/2} \end{matrix}$ (A13)

**Discussion on optoelectronic oscillator phase noise performance and prospects for integration for an optical delay (**$\tau_{D}$**) comparable to the bandpass filter delay (**$\tau_{R})$

In the case of an intra-loop phase fluctuation spectral density independent of the fibre length, the close to carrier ($\omega\tau_{D}<1$) phase noise spectral density is proportional to $1/{\left( \omega\tau_{D} \right)^{2}}$. The theoretical description remains valid as $\tau_{D}\to\tau_{R}$ but the character of the oscillator progressively evolves from a multimode time delay oscillator with properties dominated by the delay line to a classical single mode oscillator with properties dominated by the RF bandpass filter (resonator). In terms of the phase noise spectral density, the close-in phase noise of the time delay oscillator is low ${\sim1}/{\left( \omega\tau_{D} \right)^{2}}$ but features spurious sidemode resonances whereas the close-in phase noise of the classical oscillator is high ${\sim1}/{\left( \omega\tau_{R} \right)^{2}}$ but is free spurious sidemode resonances.

In the authors’ experiments, the on-resonance group delay of the RF-bandpass filter is $\tau_{R}\sim100 ns$ which equates to a waveguide delay line $\sim20 m$ in length. Implementation as an integrated waveguide (or as a compact ring resonator with the same on-resonance group delay) would require waveguide losses lower than $\sim0.20 dB m^{-1}$. Currently commercially accessible low-loss photonic integration platforms feature waveguide losses two orders of magnitude higher $\sim0.20 dB {cm}^{-1}$. Losses of $\sim0.1 dB m^{-1}$ have been demonstrated in the laboratory using specialised processes. The demonstration of fully integrated OEOs is an important milestone and there are projections that suggest that processes featuring waveguide losses of $\sim0.01 dB m^{-1}$ are feasible. However, the integrated OEOs demonstrated to date fail to leverage the delay and their phase noise performance is poor.

**Optoelectronic oscillator under proportional control: location of system poles**

The poles are located at the roots of the characteristic function (Eq. (17) main manuscript)

$\left( 1+F\left( s \right) \right)-\frac{1}{\left( 1+\tau_{R}s \right)}\exp\left( -\tau_{D}s \right)=0$ (A14)

Simple proportional control corresponds to setting$F=\kappa$. Following the method for the free oscillator described in the main text, the Lambert equation for the roots is then:

$\begin{matrix} \left( \rho+\tau_{D}s \right)\exp\left( \rho+\tau_{D}s \right)={\rho\exp\left( \rho\right)}/\left( 1+\kappa\right) \end{matrix}$ (A15)

which implies:

$\begin{matrix} s_{k}\tau_{D}= w_{k}\left( \rho\exp\left( \rho-\log\left( 1+\kappa\right) \right) \right)- \rho\end{matrix}$ (A16)

Eq. (A16) demonstrates that for$\begin{matrix} \rho\gg\left| \log\left( 1+\kappa\right) \right| \end{matrix}$ the proportional control displaces the poles further into the left half-plane substantially by the same amount $\sim-\kappa$ for small proportional gain$\left| \kappa\right|\ll1$ (see Fig. A1).

**
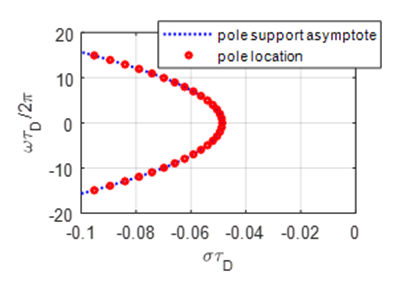
**

Fig. 1A. The predicted location of the poles of the system function of a proportionally controlled time delay oscillator in the phased locked loop configuration (${\tau_{D}}/{\tau_{R}}=300; \kappa=0.05$). All poles have been displaced further along the negative real axis substantially by the proportional gain.

**Controlled optoelectronic oscillator experiment: Bode plots & outer pole locations**

The simulated open loop bode plot for the two different sets of loop filter is presented in the main manuscript. The corresponding system pole locations of the phase locked oscillator is presented herein.

**
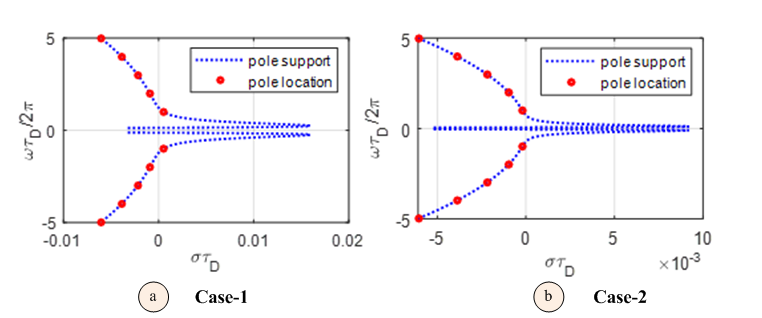
**

Fig. 2A. Phase locked oscillator outer system pole locations; (a) Case-1; modulational instability arises due to the small positive real part of the poles associated to the first adjacent sidemodes; (b) Case-2; all pole locations have a negative real part albeit slight. Modulational instability is suppressed.

**Equations used to calculate the values listed in Table 1(b):**

$\kappa=\frac{\tau_{2}}{\tau_{1}}\frac{1}{N}K_{VCO}\tau_{D}I_{CP}R_{19}$ (A17)

$\tau_{1}=R_{19}\left( C_{25}+C_{28} \right)$ (A18)

$\tau_{2}=R_{21}C_{25}$ (A19)

$\tau_{3}=R_{21}\frac{C_{25}C_{28}}{\left( C_{25}+C_{28} \right)}$ (A20)

$\tau_{4}=R_{19}C_{17}$ (A21)

$\tau_{5}=R_{14}C_{5}$ (A22)
